# No lipiodol, no beads—another transcatheter arterial chemoembolization (TACE) with fine cisplatin powder and porous gelatin particles for TACE‐naïve, multifocal, up‐to‐seven out hepatocellular carcinoma

**DOI:** 10.1002/cam4.7446

**Published:** 2024-07-17

**Authors:** Rui Sato, Michihisa Moriguchi, Atsushi Saiga, Kazuhisa Asahara, Takeshi Aramaki

**Affiliations:** ^1^ Division of Interventional Radiology Shizuoka Cancer Center Shizuoka Japan; ^2^ Molecular Gastroenterology and Hepatology Graduate School of Medical Science Kyoto Prefectural University of Medicine Kyoto Japan

**Keywords:** cisplatin, DDP‐H, gelatin, TACE, up‐to‐seven out

## Abstract

**Aim:**

The Japanese Interventional oncology group (JIVROSG) showed the efficacy and safety of nonselective transarterial chemoembolization (TACE) with fine cisplatin powder (diamminedichloroplatinum; DDP‐H) (65 mg/m^2^) and porous gelatin particles (DDP‐H TACE) without lipiodol for extensive multifocal hepatocellular carcinoma (HCC). However, there are no studies on this method following the JIVROSG study. Therefore, we aimed to evaluate the efficacy of this new DDP‐H TACE and its effect on liver function.

**Methods:**

We retrospectively reviewed the medical records of TACE‐naïve patients with multifocal HCC (Child‐Pugh class A, up‐to‐seven out, no prior history of systemic therapy) who underwent whole‐liver DDP‐H TACE between January 2006 and December 2019.

**Results:**

Sixty patients were included in this study. The median age of the patients was 71 (range, 35–88) years. The median maximum size of tumors was 26 (range, 8–184) mm; 86.7% of patients met the up‐to‐11 criteria out. The overall survival duration was 30.3 months. At the time of initial evaluation (median, 45 days), the overall response rate was 65.0%; the disease control rate was 86.7% based on the modified response evaluation criteria in solid tumors guideline. Although nine patients' liver function had deteriorated to Child‐Pugh class B at initial evaluation, six of them recovered to Child‐Pugh class A. Only three patients (5%) showed permanently impaired liver function.

**Conclusions:**

Whole‐liver DDP‐H TACE without lipiodol or beads effectively reduced tumors and preserved liver function.

## 1 INTRODUCTION

Transarterial chemoembolization (TACE) is among the guideline‐recommended choices for intermediate‐stage hepatocellular carcinoma (HCC).^1^, ^2^ Regardless of the size or number of tumors, TACE is performed only in cases of intermediate‐stage disease. Recently, the strategy has changed. The concept of TACE‐unsuitable patients is well‐established.^3^ Kudo et al.^4^ defined TACE‐unsuitability according to the following criteria: (i) unlikely to respond to TACE: multilobar type, massive or infiltrative type, simple nodular type with extranodular growth, poorly differentiated type, intrahepatic multiple disseminated nodules, or sarcomatous changes after TACE; (ii) likely to experience TACE failure/refractoriness: up‐to‐seven criteria out nodules; and (iii) likely to deteriorate to Child‐Pugh class B or C after TACE: up‐to‐seven criteria out nodules, particularly bilobar, multifocal HCC, and modified albumin‐bilirubin grade 2b. They showed that lenvatinib was better than TACE for up‐to‐seven out patients in a proof‐of‐concept study. Currently, up‐to‐seven out patients are not considered suitable for TACE. However, TACE has some advantages over systemic therapy. First, it is an intermittent treatment; the duration of side effects is limited. Second, it can cause tumor shrinkage rapidly. Since some patients achieve a complete response (CR) with a one‐time procedure, it can be considered a beneficial option.

Although we agree with the concept of TACE‐unsuitable patients,^3^ TACE in such cases usually means TACE using lipiodol (conventional TACE, cTACE). Lipiodol is a semi‐fluid substance that can flow into the surrounding portal venules and sinusoids through the peribiliary plexus and hepatic sinusoids. It has a strong embolization effect but damages liver function if super‐selective catheterization is not performed.^5^

The Japanese Interventional oncology group (JIVROSG) showed the efficacy and safety of whole‐liver nonselective TACE with fine cisplatin powder (diamminedichloroplatinum; DDP‐H) (65 mg/m^2^) and porous gelatin particles without lipiodol for extensive multifocal HCC.^6^ The overall response rate (ORR) was 25.6% based on the response evaluation criteria in solid tumors (RECIST). However, they did not evaluate changes in liver function after TACE. This method achieved relative proximal embolization even though lipiodol was not used. Therefore, liver damage due to this procedure would be less severe than that due to cTACE. Theoretically, it can help preserve liver function during treatment.^3^ Hence, the present study aimed to evaluate the efficacy and changes in liver function in patients who underwent whole‐liver DDP‐H TACE at a single center.

## 2 METHODS

### 2.1 Patient eligibility

**FIGURE 1:**
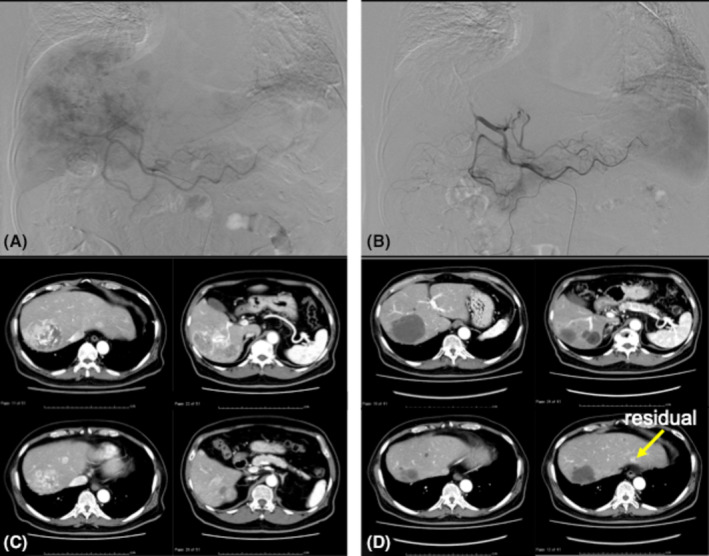
Good response case of DDP‐H TACE as per the JIVROSG 0401 protocol before (A) (C) and after (B) (D) embolization. (D) initial evaluation 1 month later.

We retrospectively reviewed the medical records of patients with HCC who underwent nonselective whole‐liver DDP‐H TACE between January 2006 and December 2019. The TACE protocol used was that adopted by the JIVROSG^6^ (Figure 1). The inclusion criteria were no prior history of TACE/hepatic arterial‐infusion chemotherapy/systemic therapy, Child‐Pugh class A, up‐to‐seven out HCC, bilobar location, and whole‐residual tumor.

### 2.2 TACE procedure

We performed the TACE procedure according to the JIVROSG 0401 protocol.^6^ After placing a microcatheter in the hepatic artery, the planned DDP‐H (IA call®; Nippon Kayaku, Tokyo, Japan) was infused (65 mg/m^2^) for 20–40 min. There were no clear criteria for reducing the cisplatin dosage; it was left to the physician's discretion. After cisplatin infusion, porous gelatin particles (Gelpart®; Nippon Kayaku, Tokyo, Japan) were infused to embolize all hepatic arteries. Occasionally, the microcatheter was advanced into the left or right hepatic artery or the distal side to avoid embolization of the cystic and right gastric arteries. The endpoint of embolization was the disappearance of tumor cells on digital subtraction angiography following the procedure (Figure 1).

### 2.3 Pre‐ and post‐TACE management

A massive intravenous infusion was necessary for preventing renal injury caused by cisplatin. According to our protocol, 1 L of normal saline was infused before TACE and 2 L of transfusion was performed after TACE on the day of TACE administration. The next day, 2 L of transfusion was performed and reduced by 500 mL each day. To prevent post‐embolization syndrome, 100 mg of hydrocortisone was given daily intravenously. Even if the patient was doing well, 6 days of hospitalization was the basic rule.

### 2.4 Evaluation of efficacy and safety

Overall survival (OS), progression‐free survival (PFS), response rate, adverse event (AE), percentage of impaired liver function, and factors associated with liver function were analyzed. The response rate was evaluated at the time of the initial computed tomography (CT) scan after TACE using the RECIST 1.1 and modified RECIST (mRECIST) criteria. Generally, TACE was repeated when a residual tumor was observed, even if it was not progressing. If repeated DDP‐H TACE was performed without progressive disease (PD), PD was defined at the time of PD after repeated DDP‐H TACE. If the strategy was changed into curative treatment such as surgery, radiofrequency ablation, and cTACE after tumor shrinkage, PD was defined at the time of PD after curative treatment. If the strategy was changed into other therapy even if it was stable disease (SD) not PD, it was a censored case at the time of images before next treatment. The reduction rate of the maximum tumor size at initial evaluation after TACE was expressed using the waterfall plot. AEs were evaluated using the common terminology criteria for adverse effects, v5.0 until first image evaluation. Two interventional radiologists evaluated treatment efficacy on CT scan. The years of experience as interventional radiologists were 25 and 10 years.

### 2.5 Statistical analysis

The OS and PFS were analyzed using Kaplan–Meier survival curves. The log‐rank test was used to assess differences in OS by initial effects of DDP‐H TACE. Logistic regression analysis was used to determine the prognostic factors associated with the deterioration in liver function. All statistical analyses were performed using the JMP17.0 (SAS Institute Inc, NC, USA).

### 2.6 Ethics

This study protocol was reviewed and approved by the Institutional Review Board in Shizuoka Cancer Center, Japan (approval number: J2023‐88‐2023‐1‐3). Patient consent was not required as this study was based on publicly available data.

## 3 RESULTS

### 3.1 Patient's background

Sixty patients were included in this study. The background data are presented in Table 1. Six patients had Barcelona Clinic Liver Cancer (BCLC)‐C status with major vascular invasion or extrahepatic metastasis (except only Physical status 1 and 2). The rate of intrahepatic metastasis was 78.3%. The proportion of up‐to‐11 criteria out patients was 86.7%. The dose of DDP‐H was reduced to 70% of the standard dose in six patients for various reasons at the physician's discretion (three cases: decreased kidney function, 1 case each: decreased physical status, leukopenia, unknown). The median number of DDP‐H TACE procedures was 2 (range, 1–8). The median number of total TACE (including cTACE and drug‐eluting bead TACE performed after the first DDP‐H TACE) in the course of treatment was 4 (range, 1–17).

**TABLE 1:** Background of patients.

	*n* = 60
Age, median (range)	71 (35–88)
Sex, Male/Female	50/10
Etiology, HBV/HCV/non viral (%)	11/24/25 (18.3%/40%/41.7%)
History of liver resection	24 (40%)
BCLC stage, B/C (%)	49/11 (81.7%/18.3%)
Child Pugh, 5/6 (%)	41/19 (68.3%/31.7%)
mALBI Grade, 1/2a/2b (%)	31/15/14 (51.7%/25%/16.7%)
ALBI score, average ± SD	−2.61 ± 0.40
Maximum tumor size, median (range)	26 mm (8‐184 mm)
Number of tumors, more than 11 (%)	47 (78.3%)
Up‐to‐11 out (%)	52 (86.7%)
Major vascular invasion (%)	5 (8.3%)
AFP, median (range)	11.1 (1.2–109,408)
Serum Creatinine (mg/dL), median (range)	0.74 (0.28–1.35)
Dose reduction of DDP‐H, (%)	6 (10%)
Replaced RHA (%)	10 (16.7%)
Total number of DDP‐H/TACE procedure, median (range)	2 (1–8)
Total number of all TACE procedure, median (range)	4 (1–17)
PS 0/1/2	53/6/1

Abbreviations: AFP, α‐fetoprotein; ALBI, albumin‐bilirubin; BCLC, Barcelona clinic liver cancer; DDP‐H, fine cisplatin powder; PS, physical status; RHA, right hepatic artery; SD, standard deviation; TACE, transcatheter arterial chemoembolization.

### 3.2 Physician's background

Eleven physicians performed this procedure. Seven of them were interventional radiologists (years of angiography experience varied between 1 and 25 years). The other four physicians were two junior surgeons and two junior gastroenterologists who rotated the Interventional Radiology Department at that time as a residency and were beginners in angiography.

### 3.3 Efficacy

**FIGURE 2:**
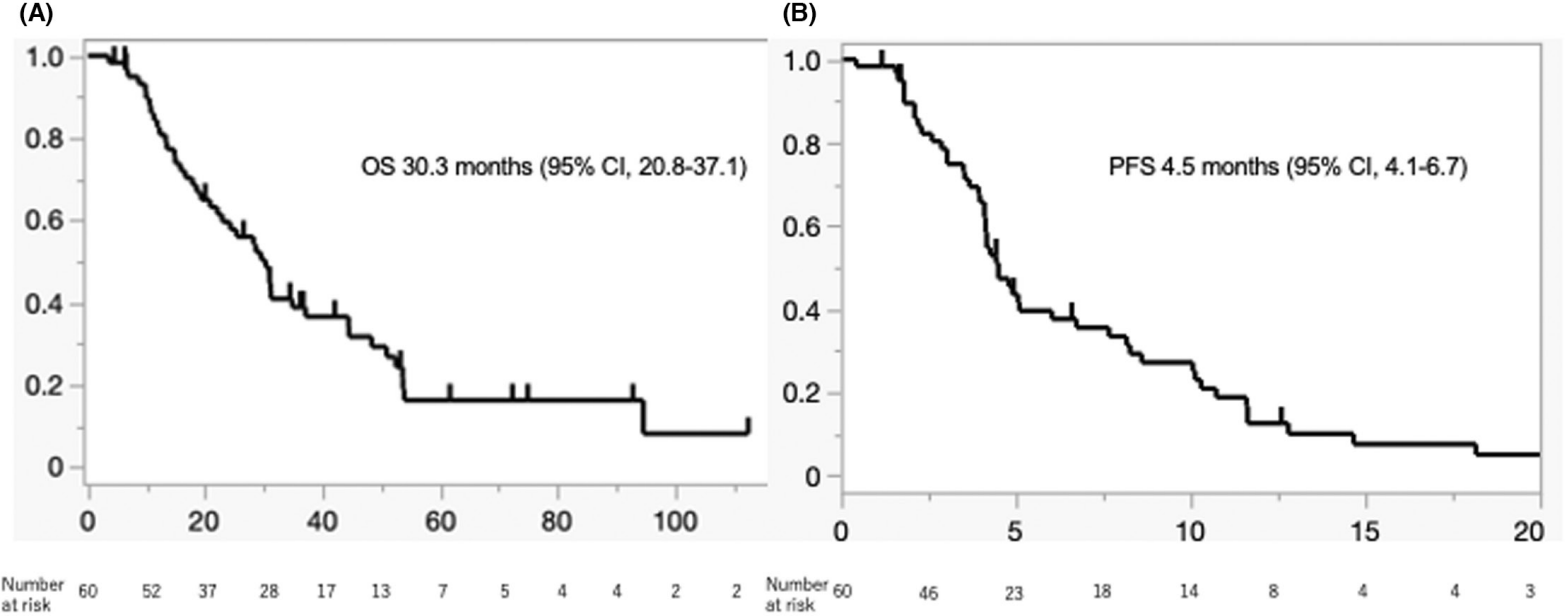
OS (A) and PFS (B) of all patients. CI, confidence interval; OS, overall survival; PFS, progression‐free survival.

**FIGURE 3:**
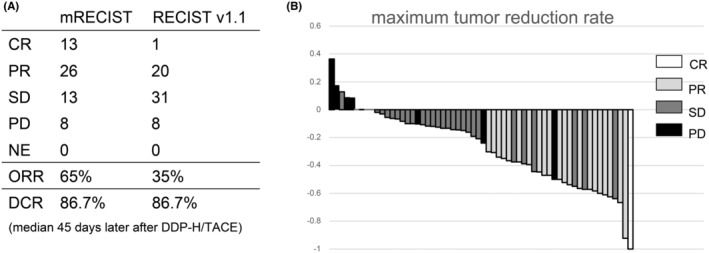
Efficacy of DDP‐H TACE (A) best response rate at initial evaluation (B) waterfall plot about maximum tumor reduction rate by RECIST. RECIST, response evaluation criteria in solid tumors.

The patients were followed up until September 2022. The median follow‐up period was 27.4 months; OS was 30.3 months (95% confidence interval [CI], 20.8–37.1), and PFS was 4.5 months (95% CI, 4.1–6.7) (Figure 2). The median evaluation period was 45 days after DDP‐H TACE on the initial contrast dynamic CT scan (arterial, portal vein, equilibrium phases). The CR rate and ORR were 21.7% and 65.0% as per the mRECIST, respectively. The CR rate and ORR were 1.7% and 35.0%, as per the RECIST, respectively. The disease control rate was 86.7%, as per both RECIST and mRECIST. The waterfall plot showed that more than 90% of cases of the maximum‐size tumor shrank, even in cases of progression (Figure 3). This showed that there were some cases wherein tumor shrinkage was achieved but new lesions resulted in PD.

The OS in the group that responded to initial treatment by RECIST (CR and PR) was 37.1 months, and that of the group that did not respond (SD and progressive disease [PD]) was 22.9 months, without significant difference (*p* = 0.09).

### 3.4 Safety

The most frequent adverse event was liver enzyme elevation (Table 2). The rate of grade 3 or 4 liver enzyme elevation was 20%. Hepatic infection and duodenal ulcer were relatively severe AEs. Both of two hepatic infection cases required percutaneous drainage. Both of two duodenal ulcer cases improved with conservative treatment with proton pump inhibitors. No grade 4 or 5 AEs were observed.

**TABLE 2:** Adverse event of DDP‐H/TACE (CTCAE v5.0).

	G1	G2	G3	G4
Fever	18	5		
Pain	6	3		
Anorexia	16			
Malaise	5			
Creatinine increased	2			
Nausea	8	4		
Hepatic infection			2	
Duodenal ulcer		2		
Esophagitis		1		
AST/ALT increased	21	9	10	2

Abbreviations: ALT, alanine aminotransferase; AST, aspartate aminotransferase; CTCAE, common terminology criteria for adverse effects; DDP‐H, diamminedichloroplatinum; TACE, transcatheter arterial chemoembolization.

The mean length of hospital stay was 8.9 days (range, 5–59 days).The percentage of hospitalizations that lasted longer than planned was 23% (14 cases). Most of the reasons were post embolization syndrome (9 cases).

The liver function in nine patients decreased to Child‐Pugh class B at the time of initial CT (median, 45 days), although six of them recovered to Child‐Pugh class A subsequently (Table 3). The prognostic factors associated with the deterioration in liver function in Child‐Pugh class B were Child‐Pugh score six and replaced right hepatic artery (RHA) on logistic regression analysis (Table 4). Two of three cases whose liver function deteriorated to Child‐Pugh B permanently were replaced RHA. The other case was liver abscess as an AE.

**TABLE 3:** Changes in liver function before and after DDP‐H/TACE at the time of initial evaluation with CT (median 45 days after DDP‐H/TACE).

	Before TACE	After TACE
CP 5/6/7≦	41/19/0	38/13/9
mALBI grade, 1/2a/2b/3	31/15/14/0	26/16/14/4
ALBI score, average ± SD	−2.61 ± 0.40	−2.49 ± 0.61

Abbreviations: ALBI, albumin bilirubin; CP, Child Pugh; DDP‐H, diamminedichloroplatinum; mALBI, modified ALBI; SD, standard deviation; TACE, transcatheter arterial chemoembolization.

**TABLE 4:** Prognostic factors associated with declined liver function to CP‐B.

	OR	95% CI	*p* Value
Age (≦70)	1.52	0.37–6.33	0.56
Sex (Male)	1.71	0.19–15.47	0.63
Non viral	3.37	0.75–15.06	0.11
Prior surgery	0.66	0.15–2.93	0.58
Up‐to‐11 out	1.27	0.13–11.80	0.83
MVI	1.47	0.14–14.99	0.74
Initial effect (SD/PD)	0.91	0.20–4.11	0.91
CP6	5.29	1.16–24.1	0.03
Replaced RHA	6	1.25–28.7	0.03

Abbreviations: CI, confidence interval; CP, Child Pugh; MVI, major vascular invasion; OR, odds ratio; PD, progressive disease; RFA, right hepatic artery; SD, stable disease.

### 3.5 Subsequent therapy

Most patients underwent subsequent treatments (96.7%) (Table 5). TACE was the most common treatment immediately after initial DDP‐H TACE (86.7%). Only two patients received systemic therapy as a second treatment (3.3%); 33.3% received systemic therapy at some point in the course of the treatment.

**TABLE 5:** Posttreatment after initial DDP‐H/TACE. (a) posttreatment immediately after DDP‐H/TACE, (b) any systemic therapy in the treatment course.

(a)	
Total	60
TACE	52
RFA combined with TACE	2
Hepatic arterial infusion chemotherapy	1
Lung resection(metastasis)	1
Systemic therapy	2
BSC	2
(b)	
Total	20
Sorafenib	13
Lenvatinib	11
Atezolizumab‐Bevacizumab	1
Regorafenib	2
Ramucirumab	2
Cabozantinib	1

Abbreviations: BSC, best supportive care; DDP‐H, diamminedichloroplatinum, RFA, radiofrequency ablation; TACE, transcatheter arterial chemoembolization.

## 4 DISCUSSION

Whole‐liver DDP‐H TACE was first introduced by Osuga et al.^6^; they reported an ORR of 25.6% as per RECIST and OS of 27.8 months. Although there was no mention of changes in liver function, the rates of liver damage (aspartate aminotransferase [AST]/alanine aminotransferase [ALT] elevation) after TACE were 28.3% (AST) and 21.7% (ALT) in grades 3 and 4, respectively, which were acceptable. Osuga et al. did not use lipiodol because it could cause biliary injury during whole‐liver embolization. However, no study has evaluated the effectiveness of DDP‐H TACE. Irie et al. examined repeated alternate infusion of cisplatin and gelatin slurry to balloon occlusion TACE (RAIB‐TACE)^7^, ^8^ without lipiodol; the ORR was 100% for giant HCC (>7 cm)^7^ and small HCC adjacent to the Glisson sheath,^8^ as per mRECIST. Their method was similar to ours. For RAIB‐TACE, dissolved cisplatin powder and gelatin slurry are used without lipiodol. Irie et al. injected the cisplatin and gelatin slurry alternately using a balloon catheter. Their procedure was more complex than ours. However, they achieved successful responses for giant HCC and HCC adjacent to the Glisson sheath, both of which are difficult to treat with cTACE.

In our study, the ORR was 65.0% and 35.0% based on mRECIST and RECIST, respectively. In most cases (more than 90%), shrinkage for the maximum tumor size was achieved after DDP‐H TACE (Figure 3). However, the OS and PFS were not favorable (30.3 months and 4.5 months, respectively). Kudo et al.^4^ found that lenvatinib was superior to cTACE in patients with up‐to‐seven out HCC as initial therapy (OS, 37.9 months vs. 21.3 months). Their population was similar to our sample. Based on the perspective of OS, our TACE was better than cTACE for up‐to‐seven out patients as initial therapy because DDP‐H TACE had a strong effect on the tumor while retaining liver function. However, TACE is only an intermittent treatment. Repeated TACE is necessary for residual tumors, even in cases achieving good tumor shrinkage. Repeated TACE can result in resistance and reduced effectiveness. This is why our OS was inferior to the lenvatinib first group in Kudo et al.'s study. Combination therapy or subsequent systemic therapy is essential to prolong the OS after DDP‐H TACE to extend the TACE interval.

The PFS was poor in our study. This is the great limitation of this procedure. Since TACE is inevitably an intermittent treatment, residual tumor will increase over time and shift to PD even with PR case. PFS cannot adequately express the effect of this treatment.

Nine patients had deteriorated to Child‐Pugh class B (15%) at the initial evaluation after TACE (median, 45 days after TACE). Although six of them recovered to Child‐Pugh class A subsequently, the liver function in three patients remained impaired permanently (5%). Saito et al. reported that the liver function in 18.5% of patients with BCLC‐B status deteriorated after super‐selective cTACE.^9^ Yasui et al. reported that the cumulative rate of deterioration in Child–Pugh class was 19.5% at 1 year after cTACE for intermediate‐stage HCC^10^; up‐to‐seven out was a significant factor for deterioration in liver function. Although our method was nonselective TACE, it resulted in less damage to liver function than the super‐selective cTACE in the study by Saito et al.,^9^ mainly because we did not use lipiodol. Lipiodol can enter the deep vessels, pass the tumor, and reach the drainage vein; it facilitates TACE but causes severe liver damage in the process. Therefore, super‐selective TACE is mandatory when using lipiodol. We only used gelatin slurry as the embolization material. Gelatin particle is relatively large and dose not reach deep into the peripheral vessels. It is also not a permanent embolization material which contains no anticancer drug. For these two reasons, we considered that DDP‐H TACE could result in less liver damage.

In our study, Child‐Pugh score six and replaced RHA were associated with a decline in liver function. Hence, patients with these features require special attention during this procedure, not to decrease liver function to Child Pugh B.

Furthermore, in the present study, it was not truly confirmed why patients with replaced RHA experienced a decline in liver function after TACE. RHA has rich communications with the left hepatic artery through the caudate arterial branch (A1) or medial segmental artery (A4), which branches from the proximal side of RHA. Using our TACE method, we embolize from the proximal side of the replaced RHA; gelatin particles flow into the A1 or A4. Embolization of the A1 or A4 can cause bile duct injury.^11^ Bile duct injury will affect liver function. We supposed that it was one possible reason for decreased liver function.

In this study, there were two grade 2 duodenal ulcer cases. It is a rare AE of selective TACE. Digital subtraction angiography after embolization revealed no apparent findings reflecting any particular problem. One case was after proton beam therapy with replaced RHA and one was post‐extended posterior segmentectomy. One possible reason was that non target embolization could occur due to such a prior treatment history, despite being not confirmed.

Although the OS in the responder group to initial treatment by RECIST (CR and PR) was much longer than that in the non‐responder group (SD and PD) (37.1 months vs. 22.9 months), the difference was not significant (*p* = 0.09). Only two patients received systemic therapy immediately after the first DDP‐H TACE. Particularly in the nonresponder group (SD and PD) at initial evaluation, only one patient received systemic therapy immediately after the first DDP‐H TACE. Most of the other patients with SD and PD underwent repeated TACE. The possible reason that fewer patients underwent systemic therapy was that only sorafenib was available commercially as systemic therapy in many cases at the time of the study. Currently, seven or eight regimens are commercially available for HCC. Several studies on the combination of TACE and systemic therapy are ongoing. DDP‐H TACE is a good candidate for this combination therapy because it can achieve a favorable response and maintain liver function. If more patients underwent subsequent systemic therapy after DDP‐H TACE or a combination of systemic therapy and DDP‐H TACE, OS would extend beyond 30.3 months.

The effect of cTACE also depends on the physician's skills.^12^ Super‐selective catheterization is necessary for cTACE^5^ but not for our DDP‐H TACE. In this study, four beginners (two junior surgeons and two junior gastroenterologists) performed this procedure and there was no difference between the experienced and inexperienced physicians. Thus, even less experienced physician can perform this procedure. This is beneficial from the point of skill standardization as the procedure is not difficult. Additionally, it aids in the education of inexperienced physicians.

This study was primarily based on data before the heyday of systemic therapy. Therefore, the results cannot be extrapolated to the current era. Recently systemic therapy has become the 1st choice for up‐to‐seven out HCC patients.^4^ In reality, DDP‐H TACE may be used as a rescue therapy in patients showing an inadequate response to systemic therapy. However, as shown in the Launch study,^13^ intrahepatic lesion control is becoming more important; DDP‐H TACE may be a good aspect in terms of combination with systemic therapy, as it can be used to shrink tumors while maintaining liver function, even if it is performed in the whole liver. DDP‐H TACE may also be the 1st choice for up‐to‐seven out patients in those who are hesitant to undergo systemic therapy such as older adults, low physical status cases, and patients who do not opt for receiving systemic therapy.

## 5 LIMITATIONS

First, this was a retrospective, single‐arm study. Therefore, randomized controlled trials are required to validate our findings. Second, the second treatment was provided at the physician's discretion; in some patients, scheduled TACE was performed, resulting in a major bias.

## 6 CONCLUSION

The whole‐liver DDP‐H TACE method effectively reduced tumors and retained liver function. It can be a feasible option in patients with intermediate HCC.

## AUTHOR CONTRIBUTIONS

**Rui Sato:** Conceptualization (lead); data curation (lead); formal analysis (lead); investigation (lead). **Michihisa Moriguchi:** Conceptualization (supporting); data curation (supporting). **Atsushi Saiga:** Data curation (supporting). **Kazuhisa Asahara:** Conceptualization (supporting); data curation (supporting). **Takeshi Aramaki:** Conceptualization (supporting); data curation (supporting).

## FUNDING INFORMATION

This study was not supported by any sponsor or funder.

## CONFLICT OF INTEREST STATEMENT

The authors have no conflicts of interest to declare about this study.

## ETHICS STATEMENT

This study was approved by the Institutional Review Board in our hospital. Formal written informed consent was not required for this retrospective chart review study according to the regulations in our country.

## Data Availability

All data generated or analyzed during this study are included in this article. Further inquiries can be directed to the corresponding author.
